# Medical Diseases of Infancy and Childhood

**Published:** 1899-06

**Authors:** 


**Medical Diseases of Infancy and Childhood.** By DAWSON WILLIAMS, M.D. Pp. xvi., 634. London: Cassell and Company, Limited. 1898.

Of the many books on the diseases of children that have appeared lately, none strikes our fancy more than that by Dr. Dawson Williams. It is a handy volume, written in clear language and not over-burdened with statistics and tables on the composition of milk. As a practical treatise in a compact form, it is without a rival, and we can confidently recommend it to those who wish to improve their knowledge on this too often neglected subject. It will not of course compare with the larger volumes that come to us across the Atlantic, but they are too large and diffuse for the ordinary reader. The illustrations are few but excellent, being mostly reproductions of photographs. The only fault we have to find is, the small amount of space given to the treatment of skin diseases. They form a large part of the cases we see in the out-patient department of hospitals for children, and from the rapidity with which they improve and get well when the proper methods of dressing are impressed on the parents, we conclude that a little more knowledge on the part of the practitioner in the poorer parts of our towns might be of use. It is, however, one thing to write and instruct, and another to learn and apply.

